# Registration rate in the Norwegian Cruciate Ligament Register: Large-volume hospitals perform better

**DOI:** 10.3109/17453674.2012.678800

**Published:** 2012-04-24

**Authors:** Karianne Ytterstad, Lars-Petter Granan, Børge Ytterstad, Kjersti Steindal, Knut Andreas Fjeldsgaard, Ove Furnes, Lars Engebretsen

**Affiliations:** ^1^The Norwegian Cruciate Ligament Register, Department of Orthopaedic Surgery, Haukeland University Hospital, Bergen; ^2^Faculty of Medicine, University of Oslo; ^3^Oslo Sports Trauma Research Center; ^4^Department of Orthopaedic Surgery, Oslo University Hospital; ^5^Department of Surgical Sciences, University of Bergen; ^6^Center for Clinical Documentation and Evaluation, Northern Norway Regional Health Authority, Tromsø; ^7^Institute of Community Health, University of Tromsø, Norway.

## Abstract

**Background and purpose:**

The Norwegian Cruciate Ligament Register (NCLR) was founded in 2004. The purpose of the NCLR is to provide representative and reliable data for future research. In this study we evaluated the development of the registration rate in the NCLR.

**Methods:**

The Norwegian Patient Register (NPR) and the electronic patient charts (EPCs) were used as reference data for public and private hospitals, respectively. Data were retrieved for all primary and revision anterior cruciate ligament (ACL) surgery during 2008–2009 in public hospitals and during 2008 in private hospitals. The NOMESCO classification of surgical procedures was used for identification of ACL surgeries. Public hospitals were divided into subgroups according to the annual number of operations in the NPR: small hospitals (< 30 operations) and large hospitals (≥ 30 operations).

**Results:**

For the 2-year data extracted from public hospitals, 2,781 and 2,393 operations met the inclusion criteria according to the NPR and the NCLR, respectively, giving an average registration rate of 86% (95% CI: 0.85–0.87). The registration rate for small public hospitals was 69% (CI: 0.65–0.73), which was significantly less than for large public hospitals (89%, CI: 0.88–0.90; p < 0.001). In 2008, private hospitals reported 548 operations to the NCLR while 637 were found in the EPCs, giving a registration rate of 86% (CI: 0.83–0.89). In that year, the registration rate for public hospitals was 86%, which was similar to that for private hospitals.

**Interpretation:**

The NCLR registration rate for the period 2008–09 was similar in both 2008 and 2009, and is satisfactory for research. There is room for improvement of registration rates, particularly in hospitals with a small volume of ACL operations.

In 2004, the incidence of ACL rupture in Norway was calculated to be 35 per 10^5^ persons per year. In the age group 16–39 years, the annual incidence was 85 per 10^5^. It has been estimated that less than 50% of these knee injuries are treated surgically, resulting in approximately 2,000 operations a year (Granan et al. 2004). The NCLR was established in 2004 (Granan et al. 2008). Reporting to the register is voluntary. The main purpose of the NCLR is to contribute to quality control, to improve the surgical cruciate ligament procedures, and to provide useful and reliable data for research. To ensure reliability, a high registration rate is essential (Granan et al. 2008). In an earlier study, the NCLR registration rate for 2006 was estimated to be 97% (Ytterstad et al. 2011). However, that study had a different study design, and it cannot be directly compared to the present study.

We evaluated the quality of registration in the NCLR by determining the registration rate of primary and revision ACL surgery over the 2-year period 2008–2009.

## Patients and methods

The NCLR achieved status as a National Medical Quality Register in 2009, and has accepted registration forms with demographic data from surgeons and patients since 2004 (Granan et al. 2008). The surgeons record former knee surgery, injury date, activity when injured, type of injury, additional injury, and technical information on the surgical method. The patient form is the validated KOOS score (Roos et al. 1998). Data were retrieved for the total amount of primary and revision ACL operations.

The Norwegian Patient Register (NPR) monitors the diagnoses and procedures carried out by public hospitals in Norway. The demographic variables are age, sex, residence, hospital, and department. Medical variables include diagnosis (ICD-10), procedure (NOMESCO – Nordic Medico-Statistical Committee, which is a Nordic classification of surgical procedures (NCSP). National language versions exist in all Nordic countries), dates of admission and discharge, and status (dead or alive) at discharge (Arthursson et al. 2005). Data were retrieved for all primary and revision ACL operations.

The electronic patient chart (EPC) contains patient information, including diagnosis and procedure codes. This information is entered directly by the medical secretary or the surgeon, and is based on recordings of the surgical procedure descriptions. Data were obtained from the EPCs of hospitals, based on a manual count of procedures.

### Data collection

2 common methods used to evaluate registration rate are (1) comparison of data in national registries with data from national patient administrative systems, and (2) comparison of data in national registries with local hospital data from question forms, surgical log books, and patient charts (Arthursson et al. 2005). Reporting to the NPR is mandatory for public hospitals only, and for private hospitals that have a business contract with the Norwegian Health Authorities. Some private hospitals operate on ACL injuries with no reimbursement from the social security system. These operations are not routinely reported to the NPR, but—according to agreement—they are still reported to the NCLR. Thus, in the present study we compared NCLR data with data from the NPR for public hospitals, while in order to avoid bias, EPC was used for comparison regarding private hospitals.

Data were collected for 2008 and 2009 (public hospitals) and for 2008 (private hospitals). Search of data in the NPR and the NCLR was performed 16 months after the end of the inclusion period, for both 2008 and 2009. Manual counting of procedures in the EPC was performed once for all 10 private hospitals, 6–8 months after the inclusion period.

The NOMESCO NCSP codes requested from the NPR were: NGE 11, NGE 15, NGE 21, NGE 25, NGE 31, NGE 35, NGE 41, NGE 45, NGE 51, NGE 55, NGE 91, and NGE 95 (KITH 2011). The data were sorted by hospital. The procedure codes include both primary and revision ACL surgery. There is no specific NCSP code for revision ACL surgery, so data on primary and revision ACL surgery could not be distinguished. All data on cruciate ligament surgery were obtained from the EPCs, not differentiating between primary and revision ACL surgery or PCL reconstruction. Distinction between ACL and PCL reconstruction was not done, since the NCLR did not have any reported PCL reconstructions reported from private hospitals for the years 2008 and 2009.

Public hospitals were divided into 2 subgroups: small hospitals with less than 30 annual operations, and large hospitals with 30 or more annual operations.

### Ethics

In accordance with the NCLR concession from the Norwegian Data Inspectorate regarding anonymity, the data retrieved from EPCs and the NPR were limited to operation counts.

## Results

### Public hospitals, 2008–2009

From all public hospitals (35 hospitals in 2008 and 30 hospitals in 2009), 2,781 cases were reported to the NPR while 2,393 cases were reported to the NCLR, making the average reporting rate 86% (95% CI: 0.85–0.87) (Table 1). The reporting rate was 86% (CI: 0.84–0.88) and 86% (CI: 0.84–0.88) in 2008 and 2009 respectively, with no significant difference (p = 0.739).

**Table 1.** Registration rate for primary and revision anterior cruciate ligament surgery in the NCLR for all public hospitals, based on operation counts for 2008 and 2009 in the Norwegian Patient Register (NPR) and the Norwegian Cruciate Ligament Register (NCLR)

Year	NCLR	NPR	Rate (%)
2008	1,184	1,373	86
2009	1,208	1,408	86
2008 and 2009	2,392	2,781	86 (95% CI: 0.85–0.87)

**Figure 1.**
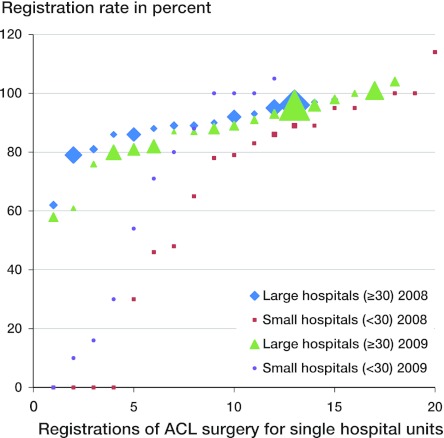
The spread in registration rate for small and large public hospitals that performed primary or revision anterior cruciate ligament surgery in 2008 and 2009. The graph contains 3 dimensions of information. The registration rate percent for each hospital unit is arranged in ascending order at the x-axis to illustrate differences between the hospital groups (dimension 1). Each point represents one hospital, and the size of each point is relative to operation volume—showing the contribution of one single hospital unit to the average registration rate. The point sizes range from 2 to 24; e.g. 0–20 operations received point size 2, 30 operations received point size 3, and 240 operations received point size 24 (dimension 2). The reporting rate percent for each hospital unit is plotted on the y-axis (dimension 3).

In 2008 and 2009 together, the reporting rate was 89% (CI: 0.88–0.90) for large hospitals and 69% (CI: 0.65–0.73) for small hospitals (p < 0.001) (Table 2). Both groups, especially small hospitals, showed considerable variation in registration rate. Some of the large hospitals with high operation volume had reporting rates that were much higher than the average reporting rate (Figure 1).

**Table 2.** Registration rate for primary and revision anterior cruciate ligament surgery in the NCLR, comparing the 2 subgroups of small and large public hospitals (< 30 and ≥ 30 annual operations) in 2008 and 2009, based on operation counts in the NPR and the NCLR

Year	Small hospitals	Large hospitals
	(NCLR/NPR)	(NCLR/NPR)
2008 **^a^**	73% (195/268)	90% (989/1,105)
2009 **^b^**	63% (108/172)	89% (1,100/1,236)
2008 and 2009	69% (303/440)	89% (2,089/2,341)

**^a ^**In 2008, there were 20 small and 15 large hospitals.

**^b ^**In 2009, there were 12 small and 18 large hospitals.

**Figure 2.**
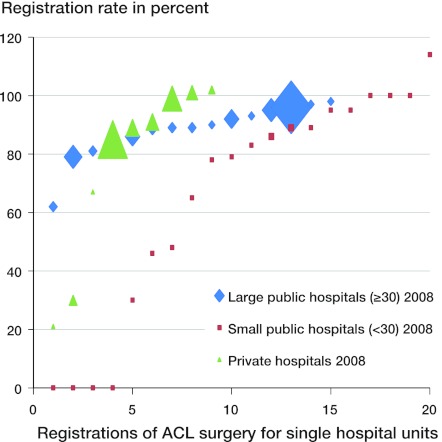
The spread in registration rate of all 44 public and private hospitals that performed cruciate ligament surgery in 2008. The graph contains 3 dimensions of information. The registration rate percent for each hospital unit is arranged in ascending order at the x-axis to represent differences between the hospital groups (dimension 1). Each point represents one hospital, and the size of each point is relative to operation volume—showing the contribution of one single hospital unit to the average registration rate. The point sizes range from 2 to 24; e.g. 0–20 operations received point size 2, 30 operations received point size 3, and 240 operations received point size 24 (dimension 2). The reporting rate percent for each hospital unit is plotted on the y-axis (dimension 3).

### Public and private hospitals, 2008

From 9 private hospitals, 637 cases were reported to the EPCs and 548 were reported to the NCLR, giving a registration rate of 86% (CI: 0.83–0.86) (Table 3). Data from one private hospital were excluded due to a gross counting error. There was no significant difference in reporting rate between private hospitals and public hospitals in 2008 (p = 0.9). Figure 2 shows the spread in registration rate of all 44 public and private hospitals that performed cruciate ligament surgery in 2008.

**Table 3.** Registration rate for primary and revision anterior cruciate ligament surgery for all 35 public and 9 private hospitals in 2008, based on operation counts in the Norwegian Cruciate Ligament Register (NCLR), the Norwegian Patient Register (NPR) and the electronic patient charts (EPCs)

Hospitals	NCLR	Denominator	Rate (%)
Public	1,184	1,373 **^a^**	86
Private	548	637 **^b^**	86

**^a ^**NPR

**^b^** EPCs

### Reporting rate

In order to test the quality of the NCLR register further, we tried to achieve a time trend comparison based on a previous study (Ytterstad et al. 2011). This comparison was based on certain procedure codes (NGE 21, 41, and 45) and on certain hospitals. The reporting rate in 2006 for 7 public hospitals was 95% (CI: 0.91–0.99) and it was 83% (CI: 0.80–0.86) for the same hospitals in the period 2008–2009. The comparison showed a decline in reporting rate (p = 0.001) (Table 4).

**Table 4.** A comparison of registration rate and operation counts for primary and revision anterior cruciate ligament surgery in 7 public hospitals in 2006, 2008, and 2009, based on the Norwegian Cruciate Ligament Register (NCLR) and the Norwegian Patient Register (NPR)

	2006 **^a^**	2008	2009
	Rate	Rate	Rate
Hospital	(NCLR/NPR)	(NCLR/NPR)	(NCLR/NPR)
1	88% (29/33)	92% (78/85)	81% (65/80)
2	100% (8/8)	100% (16/16)	100% (7/7)
3	79% (15/19)	48% (11/23)	0% (0/21)
4	100% (24/24)	93% (38/41)	98% (57/58)
5	94% (29/31)	89% (48/54)	96% (77/80)
6	160% (8/5)	30% (6/20)	30% (3/10)
7	100% (9/9)	95% (18/19)	100% (18/18)
Total	95% (122/129)	83% (215/258)	83% (227/274)

**^a ^**Results from 2006, a previous study with a different design in which hospitals were selected at random; NCPS codes differed and operations were counted for 5 months only.

## Discussion

In 2008 and 2009, public hospitals showed an acceptable and almost identical reporting rate of 86% (Table 1). The same applied for public and private hospitals in 2008 (Table 3), but comparison of private and public hospitals has obvious limitations. Firstly, we used 2 different sources of reference. Secondly, counts of ACL operations were done by staff with different degrees of training and education, which would reduce the inter-observer reliability. This may have introduced bias into the EPC data. Thirdly, a possible registration bias exists in the data from private hospitals—including PCL procedures in the data, inseparable from ACL. However, this bias would be expected to be minimal, as PCL procedures account for less than 3% of all cruciate ligament surgery (Annual Report NCLR 2009). According to the NCLR database, there were no primary or revision PCL surgeries registered in the private hospitals in 2008 (Annual Report NCLR 2009).

We found that large hospitals performed better than small hospitals, regarding registration rates. Studies have shown that less common procedures (e.g. hand surgery) and revision surgery have lower registration rates than common procedures and primary surgery (Espehaug et al. 2006). Due to unspecific NSCP codes, revisions cannot be differentiated from primary surgery. The NCLR will bring this to the attention of the NOMESCO committee.

A similar study from 2006 found a registration rate to the NCLR of 97% (Ytterstad et al. 2011). That study had limitations, however, as it only included 3 of the NCSP codes used in the present study and it investigated registration rate for a random sample of hospitals over 5 months only. However, the NCLR and NPR data obtained in 2006 could be compared to the same data from 2008–2009 taken from 7 public hospitals for procedure codes NGE 21, 41, and 45. By far the most frequent NCSP codes used are NGE 41 and 45. These 2 codes together accounted for all ACL operations in 2006, for 91% in 2008, and for 93% in 2009. Comparing these 7 hospitals for NGE 21, 41 and 45, the registration rate in 2006 was 95% compared to 83% in 2008 and 2009 (Table 4). The pointed out limitations weaken the time trend comparison, but gives rise to the concern about a possible decline in registration rate since 2006.

The Swedish Cruciate Ligament Register estimates that it has a reporting rate of greater than 90% according to a recent annual report (Annual Report SCLR 2010). The Danish Cruciate Ligament register had an increase in registration rate from 74% in 2008 to 88% in 2009 according to the annual report for 2010 (Annual Report DCLR 2010), and reporting is now mandatory. In the Finnish National Arthroplasty Register the registration rate was less than 90% in 1995, but it later increased to 95%. Reporting to this register became mandatory in 1997 (Puolakka et al. 2001).

A limitation of our study was the variation in quality of data received from the NPR. Several authors have described weaknesses in NPR reporting (Pedersen et al. 2004, Hoddevik 2005, Lofthus et al. 2005, Espehaug et al. 2006), particularly for less common procedures (Lofthus et al. 2005, Espehaug et al. 2006). Electronic databases such as EPC and NPR may contain errors arising from coding (Hoddevik 2005), data entry, transfers to other hospitals, and faulty extraction of data (Lofthus et al. 2005). Hospitals in Norway use different computer software, with no national standard. In addition, many hospitals lack official training programs for coding of procedures (Lofthus et al. 2005).

Another source of error in NPR data may be the practice of reimbursement. Diagnostic and surgical procedure codes form the basis of 40% of state financing of hospitals. Financial considerations may bias reporting and coding.

Human and systematic errors threaten the quality of data and, in our case, the registration rate. Healthcare providers and administrators at all levels must recognize that improvement of data quality is an important way to add value to the health service offered. The quality of data should be monitored routinely, with qualitative analysis of medical records, checks on data entry, and checks on the quality of abstracted data (WHO 2011).

For more in-depth analysis of registration rate, longitudinal collection of data on each patient is necessary. It is theoretically possible to link the personal ID number received from the NPR to the data in the NCLR. This linking requires permission from the Norwegian Data Inspectorate. The NCLR applied for this in 2011.

The challenges in maintaining good data quality have become obvious to us after carrying out this study. Some of these experiences were specific to this study, but they are perhaps also of general interest. Hospitals that had undergone reorganization, also with contact persons changing their positions, showed weaker reporting routines. In conversations with contact people in small hospitals, they described reduced motivation, which could be explained by the lack of feedback on revision rate, for example, because of the small number of operations annually. Perhaps one should consider this feedback from a motivational point of view as well as from a statistical standpoint. They also emphasized the importance of feedback on the quality of surgery in small hospitals, not least because of the ongoing process of centralizing surgery in large hospitals according to the premise “the greater the volume of surgery, the better the quality”.

To improve the completeness of registration in the NCLR, it is important to have good routines for communication with the local contact persons at each hospital. Moreover, one must have full knowledge of which hospitals carry out cruciate ligament surgery, so that the codebook of the NCLR is complete. The naming of hospitals in the codebook and in the NPR should be identical and should specify the place of residence.

Generally speaking, there are reasons to believe that a registry that is actively used for research will be improved by the positive attention it attracts from both hospital staff and NCLR staff. Local enthusiasm for the registry will probably be further improved in Norway when it can supply open hospital-specific data as happens in Denmark (Annual Report DCLR 2010) and Sweden (Annual Report SCLR 2010). Regarding the creation and maintenance of health registries, documentation of diagnostic improvements and treatment improvements as a result of analysis of registry data is probably the best justification for resource allocation.
